# Spatial distribution of tuberculosis in a rural region of Western Province, Papua New Guinea

**DOI:** 10.5365/wpsar.2019.10.2.001

**Published:** 2019-12-26

**Authors:** Tanya Diefenbach-Elstob, Vanina Guernier-Cambert, Bisato Gula, Robert Dowi, Daniel Pelowa, William Pomat, Catherine Rush, David Plummer, Emma McBryde, Jeffrey Warner

**Affiliations:** a College of Public Health, Medical and Veterinary Sciences, James Cook University, Townsville, QLD 4811, Australia.; b Australian Institute of Tropical Health and Medicine, James Cook University, Townsville, QLD 4811, Australia.; c Balimo District Hospital, Balimo, Western Province, Papua New Guinea.; d Papua New Guinea Institute of Medical Research, Goroka, Eastern Highlands Province, Papua New Guinea.

## Abstract

**Introduction:**

There is a high burden of tuberculosis (TB) in the Western Province, Papua New Guinea. This study aims to describe the spatial distribution of TB in the Balimo District Hospital (BDH) catchment area to identify TB patient clusters and factors associated with high rates of TB.

**Methods:**

Information about TB patients was obtained from the BDH TB patient register for the period 26 April 2013 to 25 February 2017. The locations of TB patients were mapped, and the spatial scan statistic was used to identify high- and low-rate TB clusters in the BDH catchment area.

**Results:**

A total of 1568 patients were mapped with most being from the Balimo Urban (*n* = 252), Gogodala Rural (*n* = 1010) and Bamu Rural (*n* = 295) local level government (LLG) areas. In the Gogodala region (Balimo Urban and Gogodala Rural LLGs), high-rate clusters occurred closer to the town of Balimo, while low-rate clusters were located in more remote regions. In addition, closer proximity to Balimo was a predictor of high-rate clustering.

**Discussion:**

There is heterogeneity in the distribution of TB in the Balimo region. Active case-finding activities indicated potential underdiagnosis of TB and the possibility of associated missed diagnoses of TB. The large BDH catchment area emphasizes the importance of the hospital in managing TB in this rural region.

Western Province in Papua New Guinea (PNG) has a very high burden of tuberculosis (TB) with a case notification rate of 674 per 100 000 people in 2016. (*1*) TB is known to cause a high burden of disease in Balimo and the Gogodala region of the Middle Fly District; the average reported incidence of TB at Balimo District Hospital (BDH) was 727 cases per 100 000 people per year from 2014 to 2016 for people in the combined Balimo and Gogodala local level government (LLG) areas. (*2*) Furthermore, rates of paediatric and extrapulmonary TB have been identified as very high, with 25.0% of patients aged 0–14 years, and 77.1% of patients diagnosed with extrapulmonary TB infection. (*2*)

BDH is the primary facility providing TB diagnosis and DOTS-based treatment in the Gogodala region. Other smaller health facilities, including health clinics and aid posts, can provide limited TB services such as clinical extrapulmonary TB diagnosis and treatment and pulmonary TB services when a sputum sample is not able to be transferred to the town of Balimo. (*2*) Given the high burden of TB reported at BDH, there is a need to understand the distribution of TB in the Balimo region. Such analysis will provide insight into areas with high and low rates of TB as well as evidence to support the focused delivery of TB services. This study used spatial epidemiology techniques to (1) define the catchment area of BDH, (2) identify clustering of TB in the BDH catchment area and (3) investigate factors associated with high rates of TB. The approach aimed to illustrate the local TB burden in the context of the geography of this remote region of PNG, using mapping to illustrate the results as a complement to the underlying quantitative spatial analysis.

## STUDY POPULATION AND Methods

### Study setting and patient cohort

Patient data were obtained from the BDH TB patient register, which includes all patients diagnosed and commenced on TB treatment at BDH, as described previously. (*2*) TB patients may be bacteriologically confirmed using smear microscopy or diagnosed clinically as occurs for the majority of cases in the Balimo region, in accordance with the World Health Organization (WHO) case definitions and PNG National Tuberculosis Management Protocol. (*2*-*4*) In this study, patient locations were identified as the first village recorded as a residential address for each patient. Out of 1614 TB patients registered from 26 April 2013 to 25 February 2017, 1568 were mapped after excluding patients from outside Western Province (*n* = 13) and those for whom a residential address could not be determined (*n* = 33).

### Geographic and population data

This study focused on the Balimo Urban (population 4394), Gogodala Rural (population 33 033) and Bamu Rural (population 13 432) LLG areas. In PNG, LLG areas are subdivided into rural wards and urban areas and further subdivided into census units. For this study, each patient’s location was matched to a census unit and from there to an electoral ward based primarily on PNG census data or, alternatively, on the 2012 and 2017 PNG government election polling schedules. (*5*, *6*) Instances of alternate local names were checked and confirmed locally.

Provincial, district and LLG boundary data and latitude and longitude coordinates of census units were obtained from the PNG National Statistical Office and census data. For coordinates that could not be obtained from census data, alternate sources including ArcGIS Online (Esri, Redlands, CA, USA) and a 2018 Google search were used. Population data for electoral wards used the 2011 national census figures (*7*) to describe the underlying population at risk in the cluster analyses and logistic regression. Population size was not projected to later years as ward-level population growth data were not available.

### Mapping and cluster analyses

**Figure 1:**
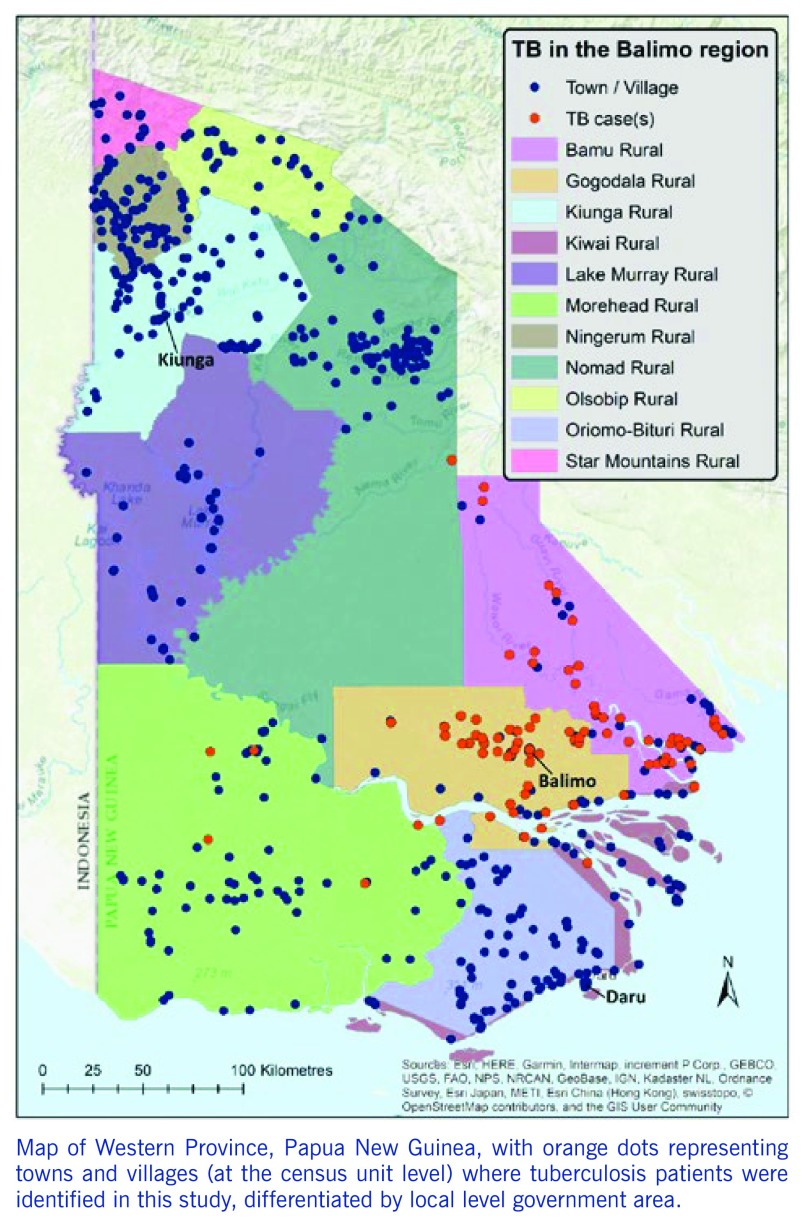
**Map of Western Province, Papua New Guinea, with orange dots representing towns and villages (at the census unit level) where tuberculosis patients were identified in this study, differentiated by local level government area.**

**Figure 2:**
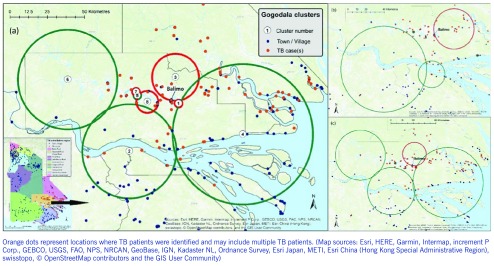
**Geographic distribution of high-rate (red circles) and low-rate (green circles) tuberculosis clusters identified in the analysis of wards in the Gogodata region in Western Province, Papua New Guinea. Cluster analyses are depicted for (a) all patients, (b) paediatric TB patients and (c) extrapulmonary TB patients.**

**Figure 3:**
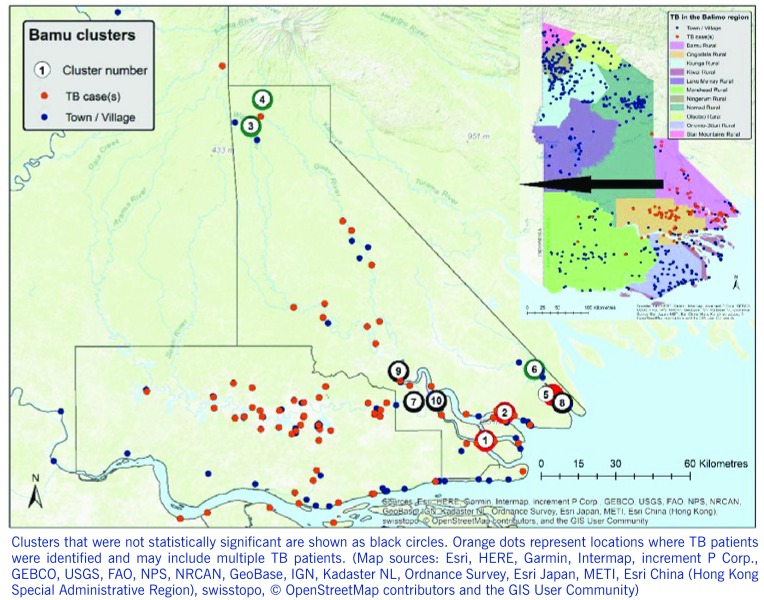
**Geographic distribution of high-rate (red circles) and low-rate (green circles) tuberculosis clusters identified in the analysis of wards in the Bamu Rural local level government area in Western Province, Papua New Guinea**

The residential locations of TB patients diagnosed at BDH were mapped to identify the BDH catchment area (i.e. the region served by the hospital as defined by the origins of TB patients who have travelled to the hospital). Mapping of residential locations was primarily based on census unit-level coordinates. However, patients from some locations were mapped based on the average coordinates of a combination of census units as the precise census unit was rarely known for these patients. Average ward coordinates were calculated using the Geographic Midpoint Calculator available in 2017. There were four locations where all census units within a ward were averaged and four locations where the averaged coordinates included several census units within a ward. Towns and villages are depicted in Fig. 1 to 3 spatially as dots as we did not have access to georeferenced boundaries at the ward level for this region of PNG.

Cluster analyses were undertaken separately for the Gogodala and Bamu regions using averaged ward-level coordinates and ward-level population data. The Gogodala region included the 39 Gogodala Rural LLG wards plus Balimo Urban LLG; the Bamu region included the 19 Bamu Rural LLG wards. (*7*) Eleven patients located within Western Province but outside the Gogodala and Bamu regions were excluded from the cluster analyses.

Cluster analyses based on paediatric and extrapulmonary TB cases were undertaken to compare clusters in these patient groups to the overall cluster analysis. These subanalyses used the same underlying population and coordinate data but with case data restricted to patient subgroups in the Gogodala region only. Age-stratified population data were not available for the wards in this region, so geographic differences in age distribution were not taken into account in the overall analysis.

The spatial scan statistic was calculated using SaTScan^TM^ (version 9.6) (SaTScan, Boston, MA., USA). (*8*)

A discrete Poisson probability model was used because occurrence of the disease is rare. (*9*, *10*) The data were scanned for areas with either high- or low-rate clusters. A circular spatial window was used, and the maximum spatial cluster size was set at the default size of 50% of the population at risk. The analyses were run with the default 999 replications with statistical significance set at *P* < 0.05. Secondary clusters that were significant were non-overlapping Gini clusters. These clusters are selected to maximize the Gini index, which is a measure of statistical dispersion, and which can provide evidence of the best non-overlapping clusters to report from one larger cluster or multiple smaller clusters. (*10*, *11*) Shapefiles depicting cluster areas were generated using SaTScan^TM^. All maps were created using ArcGIS ArcMap 10.4.1(Esri, Redlands, CA., USA) and used the World Topographic Map basemap layer provided within the ArcGIS Online package.

### Investigation of high-rate TB clusters

For wards in the Gogodala region, univariate and multivariate logistic regression were used to investigate the relationship between ward-level demographic and geographic variables and the occurrence of wards in significant high-rate TB cluster areas. Based on ward-level population data, the predictor variables included gender ratio (total males/total females), housing density (total ward population/total number of households in the ward) and distance from Balimo (distance in kilometres from the averaged Balimo coordinates to the averaged ward coordinates). Distance was calculated using the National Hurricane Center Latitude/Longitude Distance Calculator. (*12*) Statistical analyses were performed using Stata/IC version 14 (StataCorp LLC, College Station, TX., USA).

## Ethics approval

This study received local approval from the Middle Fly District Health Service and the Evangelical Church of PNG Health Service. Human research ethics approval was received from the James Cook University Human Research Ethics Committee (H6432) and the PNG Medical Research Advisory Committee (MRAC No. 17.02).

## Results

The 1568 TB patients were identified at 90 localities across Western Province. These locations, shown in **Fig. 1**, are based on census unit-level coordinates (averaged where relevant; see methods) and delineated by the LLG boundaries of Western Province. The catchment area is depicted with the majority of patients originating from the Balimo Urban (*n* = 252) and Gogodala Rural (*n* = 1010) LLG areas with a large number also in Bamu Rural LLG (*n* = 295). Eleven patients were located in other LLGs in Western Province.

[**insert** Figure 1]

### Cluster analyses

High- and low-rate TB clusters are described in **Table 1**, and depictions in **Fig. 2** (for the Gogodala region, *n* = 1262) and **Fig. 3** (for the Bamu region, *n* = 295) are based on the ward-level population and TB patient data. Cluster numbers included in **Table 1** correspond to the cluster numbers depicted in **Fig. 2** and **3**. The optimal Gini coefficients were found at 20% of the population in the Gogodala region and at 10–12% of the population in the Bamu region; paediatric TB and extrapulmonary TB subgroups were at 12% and 20%, respectively. Only clusters with less than these proportions of the population at risk were reported for each region. In the Gogodala region, high-rate clusters were generally identified closer to Balimo, while low-rate clusters were seen on the outskirts of the region (**Fig. 2**). This trend continued to be evident for the paediatric (*n* = 283) and extrapulmonary TB (*n* = 978) subgroups (**Fig. 2**). In the Bamu region, three high-rate clusters were identified in the lower regions of the Bamu and Gama Rivers; low-rate clusters were identified further along the Gama River and in the far north of the Bamu Rural LLG (**Fig. 3**).

[**insert** Figure 2]

[**insert** Figure 3]

**Table 1:** High- and low-rate tuberculosis clusters identified in wards in the Gogodala and Bamu regions of Western Province, Papua New Guinea using the spatial scan statistic

No.	Locations in cluster	Pop	Obs	Exp	RR	p
**Gogodala region ** **(Balimo Urban and Gogodala Rural LLG areas)**						
**1**	↑ **Kimama**	**704**	**162**	**23.74**	**7.68**	** < 0.01**
**2**	↓ **Lewada, Dede, ** **Konedobu, Tapila, Dewala, Pagona, Duaba**	**5854**	**20**	**197.39**	**0.09**	** < 0.01**
**3**	↑ **Bamustu, Uladu, Kewa, Kotale, Tai, Balimo Urban**	**7351**	**429**	**247.87**	**2.11**	** < 0.01**
**4**	↓ **Urio, Kenewa, Waya, Ugu, Kawiapo, Aduru, Baramula**	**6970**	**116**	**235.02**	**0.44**	** < 0.01**
**5**	↓ **Awaba**	**1646**	**6**	**55.50**	**0.10**	** < 0.01**
**6**	↓ **Ali, Makapa, Sialoa**	**4147**	**68**	**139.83**	**0.46**	** < 0.01**
**7**	↑ **Pisi**	**1119**	**73**	**37.73**	**1.99**	** < 0.01**
**8**	↑ **Ike, Yau, Aketa, Adiba, Kawito Station, Dadi**	**4801**	**212**	**161.88**	**1.37**	**0.01**
**Bamu region (Bamu Rural LLG area)**						
**1**	↑ **Sisiami**	**331**	**37**	**7.27**	**5.68**	** < 0.01**
**2**	↑ **Bamio**	**741**	**54**	**16.27**	**3.84**	** < 0.01**
**3**	↓ **Samakopa**	**1292**	**2**	**28.38**	**0.06**	** < 0.01**
**4**	↓ **Kawalasi**	**654**	**2**	**14.36**	**0.13**	** < 0.01**
**5**	↑ **Nemeti**	**229**	**15**	**5.03**	**3.09**	**0.02**
**6**	↓ **Ukusi**	**293**	**0**	**6.44**	**0.00**	**0.03**
**7**	**Garu**	**549**	**3**	**12.06**	**0.24**	**0.09**
**8**	**Ibuo**	**385**	**2**	**8.46**	**0.23**	**0.27**
**9**	**Gagoro**	**184**	**8**	**4.04**	**2.01**	**0.92**
**10**	**Miruwo**	**789**	**22**	**17.33**	**1.29**	**1.00**

LLG: local level government; Pop: population; Obs: observed cases; Exp: expected cases; RR: relative risk; TB: tuberculosis

Statistical significance was set at *P* < 0.05. High- and low-rate clusters that were significant are denoted with up- and down-arrows, respectively. The number of observed cases of TB are all those diagnosed for the cluster locations over the study period (26 April 2013 – 25 February 2017)

The logistic regression results for predictors of ward-level high-rate TB clusters are summarized in **Table 2**. In both the univariate and multivariate analyses, wards in high-rate TB clusters were associated with closer proximity to Balimo. Housing density had an odds ratio of 0.63 (95% CI: 0.34–1.20) in the univariate analysis, while the odds ratio in the multivariate analysis was 1.26 (95% CI: 0.55–2.90), suggesting confounding between housing density and distance from Balimo in this analysis.

**Table 2:** Univariate and multivariate logistic regression examining ward-level predictors of high-rate TB clustering in the Gogodala region

Predictor variables	Univariate			Multivariate	
	n	OR (95% CI)	p	OR (95% CI)	p
Gender ratio*	**40**	**0.77 ** **(0.00–242.74)**	**0.93**	**-**	**-**
**Housing density^†^**	**40**	**0.63 (0.34–1.20)**	**0.14**	**1.26 (0.55–2.90)**	**0.58**
**Distance from the town of Balimo^‡^**	**40**	**0.87 (0.79–0.95)**	** < 0.01**	**0.86 (0.77–0.96)**	** < 0.01**

CI: confidence interval; OR: odds ratio *Gender ratio is defined as total males/total females. †Housing density is defined as number of persons/number of households. ‡Distance from the town of Balimo is based on distance between average ward coordinates in kilometres.

## Discussion

This study examined the spatial distribution of TB patients diagnosed at BDH. The extensive hospital catchment area highlights the considerable distance that people travel to seek care for TB symptoms; however, the capacity to travel may help define and explain the lower case numbers in communities located further away from a health centre. In the Gogodala and Bamu regions, both high- and low-rate TB clusters were identified, illustrating the heterogeneity of reported TB burden across the region with a substantially higher TB burden evident in closer proximity to Balimo.

Most villages in the Gogodala Rural LLG had TB cases identified during the study period. Villages with no reported TB patients were predominantly located south of Balimo near the Fly River. Geographic challenges may be particularly important for people from this area as travel to either Balimo or Daru is lengthy, and fuel to travel by motorized boat to Daru is expensive. However, some patients were reported from the Gogodala region between Balimo and the Fly River, which may reflect a choice to travel to Balimo or, potentially, referral from a peripheral health facility in the region. Overall, villages with no or low rates of TB should be noted for future investigation to identify people symptomatic for TB and describe treatment-seeking practices.

In this study, low-rate clusters occurred in more remote areas, while closer proximity to Balimo was a predictor of a ward located in a high-rate TB cluster. This association is potentially linked with underdiagnosis of TB in more remote areas as less arduous travel will promote better access to care. If villages in high-rate TB clusters reflect accurate rates of TB for the region more generally, villages with low rates of TB may indicate underdiagnosis of TB and are sites where active TB investigations should be undertaken. This finding is important as other research from our group has described potential underdiagnosis of TB in the Balimo region. (*13*)

The possibility of underdiagnosis of TB was emphasized by results from the cluster analysis for the Bamu Rural LLG region. The high-rate clusters in three of these wards are the result of non-routine active case-finding activities. During an eight-day period in March 2016, 96 patients from eight villages in the Bamu Rural LLG region, including villages in these three high-rate cluster wards, were diagnosed with TB. By comparison, only 31 patients from these eight villages were diagnosed over the remainder of the study period. These diagnoses demonstrate the potential of an even higher burden of TB in remote and difficult-to-reach locations, reflecting people who may not otherwise have been diagnosed with TB. The geographically distant low-rate clusters seen in the Bamu Rural LLG likely reflect a combination of access challenges and the possibility of travel by patients to health centres other than BDH for TB care.

Other studies have described higher TB density in regions with closer proximity to urbanized areas and delayed treatment-seeking in people who travelled to a health facility by foot, while increased distance and poorer access to health facilities have been associated with diagnostic delay in some resource-limited settings. (*14*-*17*) In addition, urbanization has been associated with higher rates of TB as a result of factors such as overcrowding and increased TB transmission risk; (*18*, *19*) however, it is notable that housing density in Balimo was below average for the 40 Gogodala region wards (density of 6.9 people per household compared to an average of 7.4). Previous research in PNG has noted the importance of challenging travel in the context of TB care, including in the Gogodala region where travel is primarily by boat or by foot. (*20*, *21*) In addition, where travel by boat is possible, socioeconomic factors and affordability of fuel will play a role in the ability to travel. (*20*) Other factors, including proximity to a health facility, health worker training and local TB awareness activities have been associated with increased TB notifications. (*22*-*25*) In the Gogodala region, possible reasons for locations with high case density include the presence of an actively staffed aid post or health clinic that regularly refers presumptive TB patients or increased case-finding or awareness activities.

High rates of paediatric and extrapulmonary TB have previously been identified in the Balimo region. (*2*) Separate cluster analysis of these patient subgroups identified similar patterns to the overall distribution of TB in the Gogodala region. This finding may indicate similar TB transmission patterns across the region as well as consistency in the approach to identifying TB in a region where diagnoses are predominantly based on clinical signs and symptoms.

Awaba has the largest health centre in the Gogodala region outside of Balimo. The low-rate cluster identified in the Awaba ward is due to registration of TB patients diagnosed and started on TB treatment at the Awaba Health Centre instead of at BDH. The Awaba TB register was not available for this study, although TB incidence at the centre was estimated to be 381 cases per 100 000 people per year in a 2011 Western Province TB evaluation study. (*26*)

In our study, the 11 patients from within Western Province but outside the Gogodala and Bamu regions may be important when considering importation of TB into the Gogodala region. Seven of these patients had alternative addresses recorded within the Gogodala and Bamu regions, including two at logging camps and one at a school. This suggests mobility of people in the region, particularly in the context of education and employment, which is important when considering that schools and workplaces can be important sites of TB transmission. (*27*, *28*)

In this analysis, it was assumed that a TB patient’s first recorded address was where they were living at the time of registration. However, people with more than one address recorded may be more mobile, particularly if travelling between their residential and home villages (i.e. place of birth or family village) or workplace. Thus, some patient locations may not have reflected the location where TB infection occurred. An unknown number of TB patients were registered at smaller health facilities in the Balimo region. Although such patient numbers are likely to be low, these facilities will have influenced the analyses to an unknown extent. In addition, the TB patients identified and described here will not include Balimo-region patients diagnosed and commenced on treatment in the provincial capital of Daru. Finally, this analysis was based on population data collected in the 2011 PNG census. Thus TB rates may have been inaccurate for wards that experienced unusually high or low growth in the time before and during our study period of 2013 to 2017.

## Conclusions

This analysis provides insight into TB distribution in the BDH catchment area. The results provide baseline data about TB distribution across the region as well as targeted information that points to the need for village- and ward-specific TB investigations. In this region, TB clustering likely reflects the ease with which people can travel and seek treatment, demonstrating the importance of access to health services. However, investigation of high-rate TB clusters, as well as diagnoses resulting from targeted case-finding activities, emphasize the high potential for missed TB diagnoses in the region. The potentially substantial burden of undiagnosed TB in the extensive catchment area of BDH indicates an urgent need for active case-finding activities both to reduce TB disease burden and prevent ongoing transmission of TB in the region. This study may help focus a more targeted active TB case detection programme. Furthermore, these results emphasize the importance of targeted investment in resources and facilities in the Middle Fly District to improve and strengthen the provision of TB care in Western Province.
